# Sulfation of Chondroitin Sulfate Regulates Neuronal Morphology via Src-Family Signaling with Likely Contribution from Fyn

**DOI:** 10.3390/cells15090747

**Published:** 2026-04-22

**Authors:** Saya Kubosaka, Tadahisa Mikami, Hiroshi Kitagawa

**Affiliations:** Laboratory of Biochemistry, Kobe Pharmaceutical University, Kobe 658-8558, Japan; gd236021@st.kobepharma-u.ac.jp (S.K.); tmikami@kobepharma-u.ac.jp (T.M.)

**Keywords:** chondroitin sulfate, glycan-binding receptors, neuronal morphology, Src-family kinases

## Abstract

**Highlights:**

**What are the main findings?**

- Neuronal morphology resembling early stages of polarization is regulated by highly sulfated chondroitin sulfate (CS) preparations, including CS-D- and CS-E-enriched forms.
- CS-E-enriched substrates more effectively promote a polarization-like neuronal morphology, accompanied by enhanced Src-family kinase signaling, likely involving Fyn.

**What are the implications of the main findings?**

- Highly sulfated CS subtypes may act as extracellular cues contributing to early neuronal morphogenesis.
- These findings suggest a link between extracellular matrix sulfation and Src-family signaling in neuronal morphogenesis.

**Abstract:**

Chondroitin sulfate (CS) chains are major components of the extra- and pericellular matrix in the central nervous system (CNS), and their sulfation patterns influence CNS development and function. Highly sulfated CS preparations, including CS-D- and CS-E-enriched forms, have been shown to facilitate neurite outgrowth in cultured mouse hippocampal neurons. Notably, neurons cultured on CS-D- or CS-E-enriched substrates exhibited the following distinct morphological characteristics: CS-D promoted the extension of multiple short neurites, whereas CS-E induced the formation of a single elongated neurite with a polarization-like morphology. These features are consistent with early stages of neuronal polarization. However, the specific roles of these highly sulfated CS forms in polarization-like morphology remain unclear. In this study, we demonstrate that polarization-like morphological transitions in hippocampal neurons can be modulated on mixed CS-D/CS-E substrates by varying their ratios. Compared with CS-D-enriched substrates, CS-E-enriched substrates more effectively promoted polarization-like neuronal morphology, accompanied by enhanced activation of Src-family kinases. Furthermore, forced activation of Fyn kinase induced morphological changes resembling polarization-like features in a neuroblastoma cell line, even in the absence of CS-D/CS-E mixed substrates. In conclusion, highly sulfated CS subtypes may function as extracellular cues that regulate neuronal morphology via Src-family signaling, with likely involvement of Fyn.

## 1. Introduction

**Figure 1:**
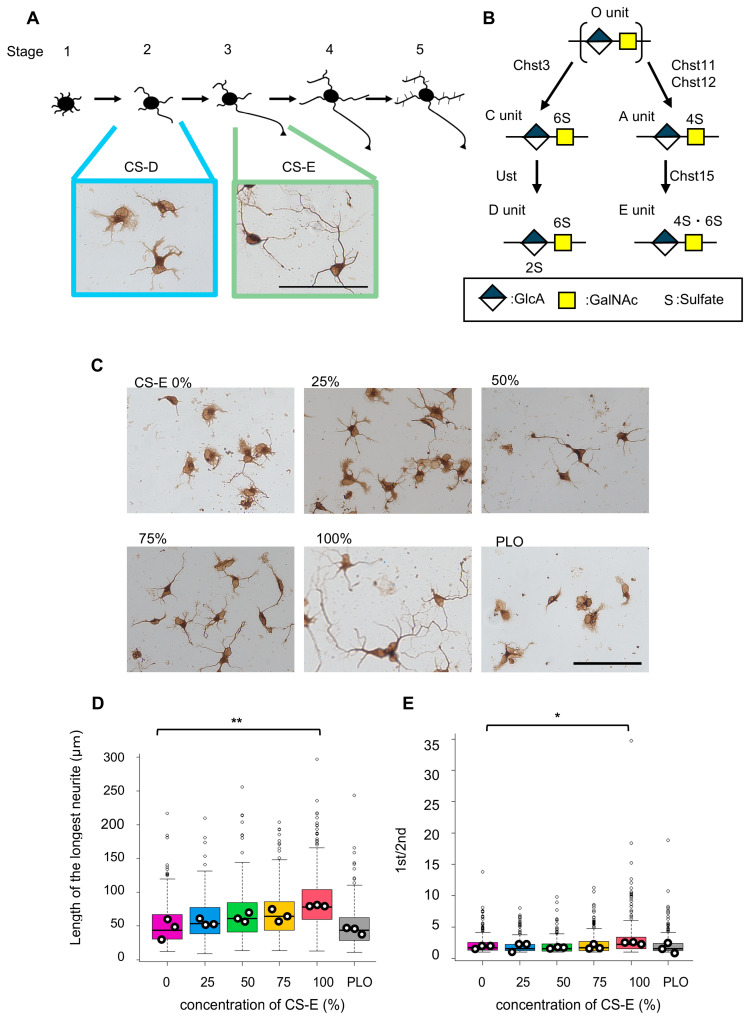
Morphological features of hippocampal neurons cultured on mixed chondroitin sulfate (CS)-D- and CS-E-enriched substrates. (**A**) Schematic illustration of the sequential stages (1–5) of neuronal polarity development in cultured hippocampal neurons (upper panels). Representative βIII-tubulin^+^ (brown) hippocampal neurons cultured on CS-D- or CS-E-enriched substrates for 48 h (lower panels) exhibit stage 2-like and stage 3-like morphologies, respectively. Scale bar = 100 µm. (**B**) Schematic diagram of the biosynthetic pathways generating CS disaccharide units. Characteristic sulfated disaccharides (A, C, D, and E) are sequentially generated from the non-sulfated precursor (O unit) by CS-specific sulfotransferases: carbohydrate sulfotransferase 11/12 (CHST11/12), carbohydrate sulfotransferase 3 (CHST3), uronyl 2-*O*-sulfotransferase (UST), and carbohydrate sulfotransferase 15 (CHST15), respectively [11]. 2S, 4S, and 6S denote 2-*O*-, 4-*O*-, and 6-*O*-sulfation, respectively. (**C**) Representative images of hippocampal neurons cultured on mixed CS-D/CS-E substrates. Poly-L-ornithine (PLO) was used as the basal control substrate prior to CS immobilization. Scale bar = 100 µm. (**D**,**E**) Quantification of the longest neurite length (**D**) and ratio of the longest to second longest neurite ((**E**), 1st/2nd) in neurons cultured under the conditions shown in (**C**). Data distributions of individual cells are presented as box plots (boxes indicate the interquartile range [IQR], whiskers represent 1.5 × IQR, horizontal lines indicate the median, and small dots represent outliers). Larger dots represent the mean of each independent culture. A total of 100 neurons were analyzed per culture, with three independent cultures per condition. Statistical analyses were performed using mean values from independent cultures. * *p* < 0.05 vs. 0% CS-E; ** *p* < 0.01 vs. 0% CS-E (one-way ANOVA followed by Dunnett’s post hoc test).

Neurons are highly polarized cells with distinct morphological and functional domains, typically consisting of a single elongated axon and multiple shorter dendrites. These structures play complementary roles in neuronal communication—axons transmit signals, whereas dendrites receive them. Therefore, the establishment of neuronal polarity is essential for proper central nervous system (CNS) development and function. Over the past few decades, extensive research has aimed to elucidate the fundamental mechanisms underlying neuronal polarity, with dissociated hippocampal neuron culture systems serving as key experimental models [1,2]. As illustrated in Figure 1A, in vitro differentiation of cultured hippocampal neurons progresses through a well-characterized sequence of five stages [1,2]. Shortly after plating, neurons appear as spherical cells with filopodia (stage 1). They subsequently extend multiple minor neurites that repeatedly elongate and retract (stage 2). Thereafter, one of these neurites undergoes rapid growth and differentiates into an axon, marking the onset of morphological polarization (stage 3). The remaining neurites gradually adopt dendritic identity (stage 4), and functional neuronal networks are established after approximately 7 days in culture (stage 5). Although our understanding of intracellular signaling networks governing neuronal polarity has advanced, research on the environmental cues that modulate these processes remains limited [3,4,5].

To date, classical extracellular cues such as laminin, Netrin-1, brain-derived neurotrophic factor (BDNF), and Wnt proteins have been shown to induce neuronal polarization through receptor-mediated signaling pathways [3,4,6,7]. In addition to these proteinaceous ligands, the extra- and pericellular environment of the CNS is rich in glycoconjugates. Among these, chondroitin sulfate (CS) is a representative sulfated glycosaminoglycan (GAG) that covalently attaches to core proteins to form CS proteoglycans (CSPGs). Although CSPGs were historically regarded as passive structural scaffolds, accumulating evidence indicates that they actively participate in diverse developmental processes, including neuronal migration, differentiation, and survival [8,9,10]. These functions are largely attributed to the sulfation patterns of CS chains [11,12]. However, whether CS itself regulates neuronal polarization in a sulfation-dependent manner remains unclear.

CS GAGs comprise a linear polysaccharide backbone composed of repeating disaccharide units of glucuronic acid (GlcA) and *N*-acetylgalactosamine (GalNAc), with structural diversity generated by CS-specific sulfotransferases (Figure 1B). Accordingly, CS chains can be classified into subtypes based on their disaccharide composition [11]. In the mammalian CNS, CS chains are predominantly composed of monosulfated disaccharide units A and C. Although present at low abundance (<1%), disulfated disaccharide units D and E are also detectable in brain-derived CS preparations [13]. Developmental studies have demonstrated that CS sulfation patterns are dynamically regulated, exhibiting distinct temporal and regional distributions of disaccharide units, including D and E units. These observations suggest that specific CS subtypes may contribute to stage-dependent regulation of neuronal differentiation and circuit formation [13,14,15]. Low-sulfated CS subtypes enriched in A and C units (CS-A and CS-C) are generally non-permissive or only weakly permissive for neurite outgrowth [16,17]. In contrast, highly sulfated CS preparations, such as CS-D- and CS-E-enriched forms, characterized by predominance of D and E units, respectively, significantly promote neurite outgrowth [17,18,19]. Notably, hippocampal neurons cultured on CS-D- and CS-E-enriched substrates exhibit distinct morphologies resembling stage 2-like and stage 3-like polarization states, respectively (Figure 1A). These recurring patterns support the hypothesis that highly sulfated CS chains act as extracellular cues influencing early neuronal morphogenesis.

In the present study, we investigated the roles of highly sulfated CS, including CS-D- and CS-E-enriched preparations, in regulating neuronal morphology associated with early stages of polarization in cultured neurons. Furthermore, we examined the involvement of Src-family kinases, including Fyn, which have been identified as intracellular signaling molecules responsive to highly sulfated CS [20,21], and assessed how their modulation correlates with polarization-like neuronal morphology. Our findings provide new insights into the contribution of CS sulfation patterns to neuronal morphogenesis.

## 2. Materials and Methods

### 2.1. Materials

CS preparations and related reagents used in this study were obtained from Seikagaku Corp. (Tokyo, Japan). CS-D derived from shark cartilage (average molecular mass 30 kDa, #400676) and CS-E derived from squid cartilage (average molecular mass 70 kDa, #400678) are commercially supplied as highly sulfated preparations enriched in their respective disaccharide units and are therefore referred to as CS-D- and CS-E-enriched preparations, respectively. Standard unsaturated CS disaccharides (#400571), including O [GlcA-GalNAc], A [GlcA-GalNAc(4-sulfate)], C [GlcA-GalNAc(6-sulfate)], D [GlcA(2-sulfate)-GalNAc(6-sulfate)], and E [GlcA-GalNAc(4,6-disulfate)], were used to identify and quantify CS disaccharide composition. For depolymerization of CS chains prior to disaccharide analysis, *Proteus vulgaris* chondroitinase ABC (ChABC; EC 4.2.2.4, #100330) was used. The disaccharide composition of CS-D- and CS-E-enriched preparations is summarized in Table 1.

Poly-L-ornithine (PLO, 30,000–70,000 Da, #P4957), DNase I (#D5025), and ovalbumin (#A5503) were purchased from Sigma-Aldrich (St. Louis, MO, USA). 4-Amino-5-(4-chlorophenyl)-7-(t-butyl)pyrazolo [3,4-d]pyrimidine (PP2; #S7008) was purchased from Selleck Biotechnology (Yokohama, Japan). Anti-βIII-tubulin (Tuj-1, #66375-1-Ig), anti-heat shock protein 90 (HSP90, #13171-1-AP), and anti-DYKDDDDK tag (#20543-1-AP) antibodies were purchased from Proteintech (Rosemond, IL, USA). Anti-phosphorylated neurofilament H antibody (SMI31, #801602) was purchased from BioLegend (San Diego, CA, USA). Anti-phospho-Src family (tyrosine 416 [Tyr416]) (D49G4, #6943) and anti-Fyn (#4023) antibodies were purchased from Cell Signaling Technology (Danvers, MA, USA). Alexa Fluor-labeled anti-green fluorescent protein (GFP) antibody (rat IgG, RQ2, #D153-A48) was purchased from MBL (Tokyo, Japan). C57BL/6J mice were obtained from CLEA Japan, Inc. (Tokyo, Japan). The mouse neuroblastoma cell line Neuro 2a (N2a, IFO50081) was obtained from the Japanese Collection of Research Bioresources (JCRBs) Cell Bank, National Institutes of Biomedical Innovation, Health and Nutrition (NIBNs, Osaka, Japan).

### 2.2. Plasmids

Primer sequences used for plasmid construction are listed in Table 2. Full-length human wild-type (WT) Fyn complementary DNA (cDNA) was amplified by polymerase chain reaction (PCR) using an in-house HeLa cell library as a template, with forward and reverse primers containing EcoRI and BamHI recognition sites, respectively. The forward primer included a Kozak consensus sequence [22] to enhance translational efficiency. The PCR product was subcloned into the p3xFLAG-CMV™-14 vector (#E7908, Sigma-Aldrich) via EcoRI and BamHI sites to generate p3xFLAG/Fyn-WT. Construct fidelity was confirmed by DNA sequencing.

Expression plasmids encoding constitutively active (CA) and dominant-negative (DN) forms of Fyn [23,24] were generated using a two-step PCR-based mutagenesis approach. Briefly, two overlapping fragments were amplified using p3xFLAG/Fyn-WT as a template and primer sets containing internal mutagenic (IM) sequences. Full-length variant cDNAs were subsequently generated by overlap extension PCR and subcloned into p3xFLAG-CMV™-14 to produce p3xFLAG/Fyn-CA and p3xFLAG/Fyn-DN. All constructs were verified by DNA sequencing. pCAG-yellow fluorescent protein (YFP, #11180) was obtained from Addgene (Watertown, MA, USA).

### 2.3. Primary Neuronal Cell Culture

Primary hippocampal neuron cultures were prepared as previously described [17,18,19,20,21], with minor modifications. Briefly, 8-well chamber slides (Nunc™ Lab-Tek™ Chamber Slide System, Thermo Fisher Scientific, Waltham, MA, USA) were pre-coated with poly-L-ornithine (PLO, 1.5 μg/mL) and subsequently overlaid with aqueous solutions of CS-D- and CS-E-enriched preparations (5.0 µg/mL) at defined mixing ratios. Pregnant WT (C57BL/6J) mice were euthanized on embryonic day 16.5 (E16.5), and embryos were rapidly dissected. Hippocampi were isolated and dissociated using 0.25% trypsin (Gibco™, #27250018; Thermo Fisher Scientific) and 0.004% DNase I. Cells were resuspended in Neurobasal Plus™ medium (#A35829-01; Thermo Fisher Scientific) supplemented with B27 Plus (1×, #A35828-01; Thermo Fisher Scientific), 5 mM GlutaMAX™ I (#35050-061; Thermo Fisher Scientific), 0.1% ovalbumin, and penicillin (100 µg/mL)–streptomycin (100 U/mL) (#26253-84; Nacalai Tesque, Kyoto, Japan). Cells were plated at a density of 1.5 × 10^4^ cells/cm^2^ onto CS-coated wells and cultured for 30–48 h at 37 °C in 5% CO_2_.

For inhibition of Src-family kinases, PP2 (0.5–10 μM) was added 2 h after plating. To block contactin-1 (CNTN-1) and integrin αvβ3 [20,21], a CNTN-1 neutralizing antibody (5 µg/mL, #AF904; R&D Systems, Minneapolis, MN, USA) and cyclic RGD peptide (cyclo, 30 µM, #4304-v; Peptide Institute Inc., Osaka, Japan) were used, respectively.

### 2.4. Neuroblastoma Culture

Neuro-2a (N2a) cells were cultured in Dulbecco’s modified Eagle’s medium (#043-30085; Fujifilm Wako, Osaka, Japan) supplemented with 10% fetal bovine serum. N2a cells stably expressed mouse CNTN-1, hereafter referred to as N2a/CNTN-1, and were generated by transfection with pCMV/CNTN-1 [20]. Clonal cell lines were selected and maintained in the presence of 800 μg/mL G418 (#09380-86; Nacalai Tesque). To assess Src/Fyn activation induced by highly sulfated CS, N2a/CNTN-1 cells (1.1 × 10^5^ cells/cm^2^) were seeded onto PLO-precoated 4-well plates in serum-free medium (Neurobasal Plus™ supplemented with B27 Plus [1x]). After 2 h at 37 °C, cells were treated with CS-D- or CS-E-enriched preparations (50 μg/mL) for 6 h.

Co-expression of YFP and Fyn kinase variants (WT, CA, or DN) was achieved by transient transfection using the Neon™ electroporation system (Thermo Fisher Scientific), according to the manufacturer’s instructions. For morphometric analysis, electroporated cells were plated at a density of 1.5 × 10^4^ cells/cm^2^ on the PLO-precoated substrates and cultured for 30–48 h in the same conditions used for primary neurons.

### 2.5. Immunocytochemistry

Hippocampal neurons and N2a-derived cells were fixed with 4% paraformaldehyde in phosphate-buffered saline (PBS), permeabilized, and blocked with 0.2% Triton X-100 in PBS containing 3% bovine serum albumin (#015-27053; Fujifilm Wako). Cells were incubated with anti-βIII-tubulin (Tuj-1; 1:1000), followed by a biotinylated secondary antibody (#BA-1300; Vector Laboratories, Burlingame, CA, USA) and the VECTASTAIN ABC kit (#PK-4000; Vector Laboratories). Signals were visualized using a Peroxidase DAB staining kit (#25985-50; Nacalai Tesque). For immunofluorescence, cells were incubated with anti-Tuj-1 (1:1000) together with either anti-phosphorylated neurofilament H (SMI31; 5 µg/mL) or anti-phospho-Src family (Tyr416; 1:100). Appropriate Alexa Fluor-conjugated secondary antibodies were applied, and nuclei were counterstained with NucBlue™ Fixed Cell Stain ReadyProbes™ reagent (4′,6-diamidino-2-phenylindole (DAPI), #R37606; Thermo Fisher Scientific). To enhance YFP signal detection, an Alexa Fluor-labeled anti-GFP antibody (1:500) was used.

### 2.6. Morphometric Analysis

Bright-field and fluorescence images were acquired using a BZ-800 all-in-one fluorescence microscope (KEYENCE, Osaka, Japan). For morphometric analysis, randomly selected neurons cells with at least one neurite longer than the cell body diameter were analyzed. For each neuronal cell, the lengths of the longest and second longest neurites were measured using ImageJ software (version 1.53t), and their ratio was calculated as the polarization-like morphology index. In selective experiments, the relative fluorescence intensity of phosphorylated neurofilament H (SMI31) in the longest neurite compared with that in the second longest neurite was quantified, and the ratio was defined as the SMI31 asymmetry index. Similarly, the relative fluorescence intensity of phosphorylated Src-family kinase (Tyr416; pY416) in the longest neurite compared with that in the second longest neurite (pY416 asymmetry index) was quantified as described previously [25]. Only neurons without obvious signs of degeneration were included in the analysis.

### 2.7. Quantitative PCR (qPCR)

Total RNA was extracted using Sepasol-RNA-I Super G reagent (#09379-97; Nacalai Tesque). RNA samples were treated with RQ1 RNase-free DNase (#M6101; Promega, Madison, WI, USA) and used for cDNA synthesis. qPCR was performed using FastStart Essential DNA Green Master Mix (#06924204001; Roche Applied Science, Penzberg, Germany) according to the manufacturer’s instructions. Primer sequences are listed in Table 3. Gene expression levels were normalized to glyceraldehyde 3-phosphate dehydrogenase (*Gapdh*).

### 2.8. Immunoblotting

**Figure 2:**
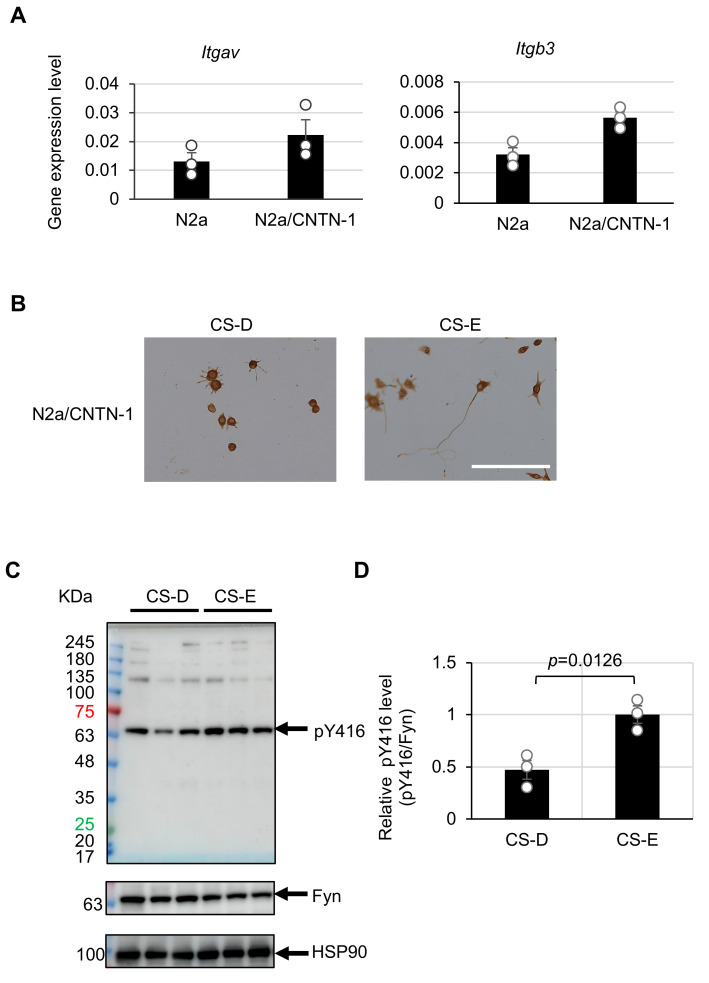
Responsiveness of Neuro-2a/contactin-1 (N2a/CNTN-1) cells to chondroitin sulfate (CS)-D- and CS-E-enriched preparations. (**A**) Quantification of *Itgav* and *Itgb3* transcripts (normalized to glyceraldehyde 3-phosphate dehydrogenase) in N2a and N2a/CNTN-1 cells. Data are presented as mean ± standard error of the mean (SEM) from three independent experiments (*n* = 3). (**B**) Representative images of N2a/CNTN-1 cells cultured on CS-D- or CS-E-enriched substrates for 48 h. Scale bar = 100 µm. (**C**) Phosphorylation levels of Src-family kinases (with likely contribution from Fyn) in N2a/CNTN-1 cells following 6 h of treatment with CS-D or CS-E, analyzed by Western blotting with antibodies against Src-family kinases (Tyr416; pY416) and Fyn. HSP90 was used as a loading control. (**D**) Relative pY416 levels (pY416/Fyn) quantified by densitometric analysis of the blots in (**C**). Data are presented as mean ± SEM from three independent experiments (*n* = 3). Statistical significance was determined using Student’s *t*-test (*p* = 0.0126).

To assess Src-family kinase activation in response to CS treatment, N2a/CNTN-1 cells were treated with CS-D- or CS-E-enriched preparations (50 μg/mL) for 6 h prior to lysis, as described in Section 2.4. Cultured cells were lysed in radioimmunoprecipitation assay buffer (#182-02451; Fujifilm Wako) supplemented with 1 mM sodium orthovanadate (#P0758S; New England Biolabs, Ipswich, MA, USA) and a protease inhibitor cocktail (#04080-11; Nacalai Tesque). Cell lysates were incubated on ice for 30 min and centrifuged at 20,000× *g* for 10 min at 4 °C. Supernatants were mixed with Bolt™ LDS sample buffer (#B0007; Thermo Fisher Scientific) and Bolt™ sample reducing agent (#B0009; Thermo Fisher Scientific) and heated at 70 °C for 10 min. Equal amounts of protein (11.25 μg per lane) were separated on Bolt™ 4–12% Bis-Tris Plus WedgeWell™ gels (#NW04127BOX; Thermo Fisher Scientific) and transferred onto Amersham™ Hybond™ polyvinylidene fluoride membranes (#10600023; Cytiva, Uppsala, Sweden) using a tank blotting system. Membranes were blocked with Blocking One-P (#05999-84; Nacalai Tesque) and incubated overnight at 4 °C with primary antibodies against phospho-Src family Tyr416 (1:1000), Fyn (1:1000), DYKDDDDK tag (1:1000), or HSP90 (1:15,000) diluted in Signal Enhancer HIKARI Solution A (#02270-81; Nacalai Tesque). Membranes were then incubated with horseradish peroxidase-conjugated anti-rabbit IgG (1:10,000, Cytiva) diluted in Signal Enhancer HIKARI Solution B (#02297-64; Nacalai Tesque). Signals were detected using Chemi-Lumi One Ultra (#11644-40; Nacalai Tesque) according to the manufacturer’s instructions. Chemiluminescent images were acquired using an Amersham™ ImageQuant 800 and analyzed with Amersham™ ImageQuant TL (Ver. 8.1; Cytiva). Uncropped blots with corresponding to Figure 2C and Figure S3 are provided in Figure S1.

### 2.9. Disaccharide Composition of CS Chains

CS-D- and CS-E-enriched preparations were digested with ChABC (5 mIU) at 37 °C for 2 h. The resulting disaccharides were derivatized with 2-aminobenzamide and analyzed by anion-exchange high-performance liquid chromatography (HPLC) using a YMC-Pack PA-G column (4.6 mm × 250 mm, #PG12S05-2546WT; YMC, Kyoto, Japan) [26]. CS disaccharides were identified and quantified by comparison with authentic standards corresponding to unsaturated disaccharides derived from O, A, C, D, and E units.

### 2.10. Statistical Analyses

All values are presented as the mean ± standard error of the mean from at least three independent experiments. Statistical analyses and data visualization were performed using EZR (version 1.68, Saitama Medical Center, Jichi Medical University, Saitama, Japan), a graphical user interface for R [27]. For analyses involving multiple cells within a single experiment, mean values from independent experiments were used as biological replicates. Data distributions are presented as box plots or bar graphs, with overlaid points representing individual observations or mean values from independent experiments, as appropriate. Homogeneity of variance was assessed prior to testing. Comparisons between two groups were performed using Student’s *t*-test. For multiple group comparisons, one-way analysis of variance (ANOVA) followed by Dunnett’s post hoc test was used. Statistical significance was defined as *p* < 0.05.

## 3. Results

### 3.1. Neuronal Morphological Transition of Hippocampal Neurons on Mixed CS-D- and CS-E-Enriched Substrates

Using our previously established assay for CS-mediated neurite outgrowth in culture [17,18,19], we obtained highly reproducible results. Consistent with our earlier findings, hippocampal neurons cultured on CS-D-enriched substrates exhibited extensive soma spreading with multiple neurites, whereas those cultured on CS-E-enriched substrates displayed a rounder soma with limited spreading and a single elongated neurite with a polarization-like morphology (Figure 1A). These distinct morphologies resembled stage 2-like and stage 3-like states, respectively, observed during neuronal polarity development, enabling assessment of whether highly sulfated CS chains contribute to early morphological transitions. Hereafter, CS-D- and CS-E-enriched preparations are referred to as “CS-D” and “CS-E,” respectively.

To test this hypothesis, we prepared culture substrates containing mixtures of CS-D and CS-E (CS-D/CS-E mixed substrates). Substrates were first precoated with poly-L-ornithine (PLO), followed by coating with highly sulfated CS polymers at varying CS-D:CS-E ratios, while maintaining a constant total CS concentration (5 µg/mL). Previous studies have shown that low-sulfated CS subtypes (CS-A and CS-C) exhibit minimal neurite outgrowth-promoting activity, comparable to that observed on PLO-coated substrates [17,20,21]. Because PLO is widely used as a basal substrate and facilitates CS immobilization via electrostatic interactions, it was used as the control condition. Dissociated hippocampal neurons were plated at low density on CS-D/CS-E mixed substrates and cultured for 48 h. Following immunostaining, neurite morphology was analyzed at the single cell level. As the longest neurite in cultured hippocampal neurons typically corresponds to the future axon [1,2,28], neuronal polarization was quantified using the following two parameters: (1) the length of the longest neurite and (2) the ratio of the longest neurite to second longest neurite.

As shown in Figure 1C, neuronal morphology varied depending on the CS-D:CS-E ratio. Quantitative analysis demonstrated that the length of the longest neurite increased significantly with increasing proportions of CS-E (Figure 1D). Similarly, the ratio of the longest to second longest neurite increased and was significantly higher on 100% CS-E compared with CS-D (0% CS-E) (Figure 1E). These results indicate that increasing CS-E content promotes neurite asymmetry characteristic of a stage 3-like morphological transition associated with early neuronal polarization.

To further assess whether the elongated neurite under CS-E-enriched conditions exhibits axon-like characteristics, immunocytochemical analysis was performed using the axonal marker SMI31, which recognizes phosphorylated neurofilament H (Figure S2). In neurons cultured on CS-E-enriched substrates, the longest neurite frequently showed stronger SMI31 immunoreactivity compared with other neurites, whereas such preferential localization was less apparent under CS-D-enriched conditions (Figure S2A). Consistent with this observation, quantitative analysis revealed that the SMI31 asymmetry index, defined as the fluorescence intensity ratio between the longest and second-longest neurites, was significantly higher under CS-E-enriched conditions than under CS-D-enriched conditions (Figure S2B). These findings further support the interpretation that CS-E-enriched conditions promote early axon-like morphological features.

### 3.2. Differential Src-Family Kinase Activation by CS-D and CS-E in Neuro-2a/Contactin-1 (N2a/CNTN-1) Cells

We previously demonstrated that CS-D- and CS-E-mediated neurite outgrowth is facilitated by their function as extracellular signaling ligands that bind to specific neuronal cell-surface CS-binding receptors and trigger intracellular signaling cascades [11,29]. CNTN-1, a cell adhesion molecule of the immunoglobulin superfamily, was identified as a receptor mediating CS-E-dependent neurite outgrowth [20]. In contrast, CS-D-dependent neurite outgrowth has been linked to signaling via the extracellular matrix receptor integrin αVβ3 [21]. These findings suggest that the polarization-like morphological transition observed on mixed CS-D/CS-E substrates may be influenced, at least in part, by the balance between intracellular signaling pathways downstream of CNTN-1 and integrin αVβ3. Notably, these pathways converge on Src-family kinases, likely involving Fyn [20,21]. To extend our observations from primary hippocampal neurons and enable more controlled mechanistic analyses, we employed a Neuro-2a (N2a) neuroblastoma cell line as a complementary model. To specifically compare the ability of CS-D and CS-E to activate Src-family kinases, we used a stable N2a cell line overexpressing CNTN-1 (N2a/CNTN-1).

Parental N2a cells lack endogenous CNTN-1 expression but acquire responsiveness to CS-E upon CNTN-1 expression [20]. Consistent with this, N2a/CNTN-1 cells expressed mRNA for integrin subunits, *Itgav* and *Itgb3* (Figure 2A). When cultured on CS-D- or CS-E-enriched substrates, these cells exhibited neurite outgrowth patterns and morphologies comparable to those of primary hippocampal neurons (Figure 2B), supporting their suitability for quantitative analysis. Src-family kinase activation was evaluated using an antibody recognizing phosphorylation at Tyr416 (pY416). Western blot analysis showed that treatment with CS-E resulted in a significantly higher pY416 signal in N2a/CNTN-1 cells than CS-D (Figure 2C,D), indicating that CS-E more effectively activates Src-family kinases. These findings suggest that enhanced Src-family activation in response to CS-E may contribute to the induction of a morphology characteristic of early neuronal polarization.

**Figure 3:**
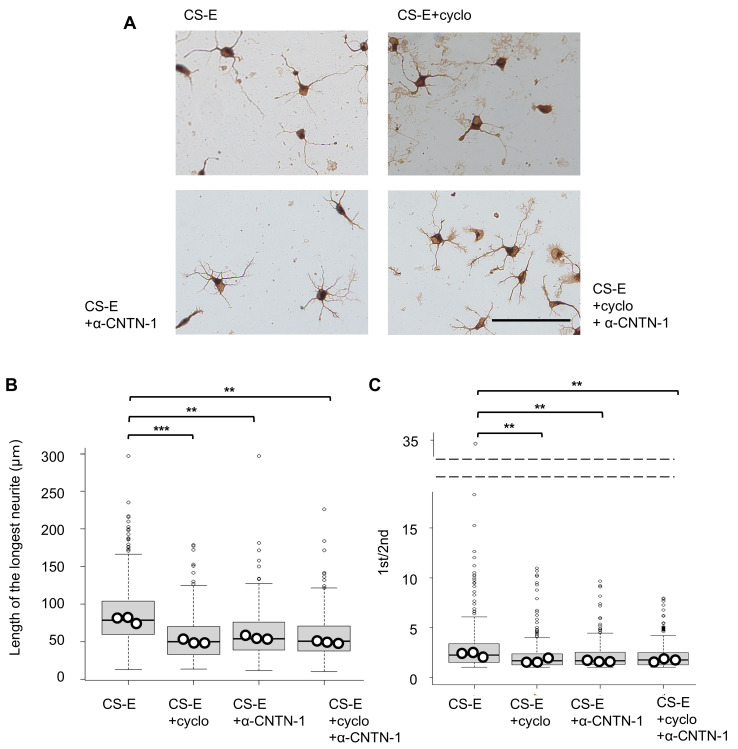
Involvement of neuronal integrin αvβ3 in chondroitin sulfate (CS)-E-mediated polarization-like neuronal morphology. (**A**) Representative images of hippocampal neurons cultured for 48 h on CS-E-enriched substrates in the presence of a CNTN-1 neutralizing antibody (5 µg/mL) and/or an αvβ3-specific antagonistic peptide (cyclo; 30 µM). Scale bar = 100 µm. (**B**,**C**) Quantification of the longest neurite length (**B**) and the ratio of the longest to second longest neurite ((**C**); 1st/2nd) in neurons cultured under the conditions shown in (**A**). Data distributions of individual cells are presented as box plots (boxes indicate the interquartile range [IQR], whiskers represent 1.5 × IQR, horizontal lines indicate the median, and small dots represent outliers). Larger dots represent the mean value from each independent culture. A total of 100 neurons were analyzed per culture, with three independent cultures per condition. Statistical analyses were performed using mean values from independent cultures. ** *p* < 0.01 vs. CS-E; *** *p* < 0.001 vs. CS-E (one-way ANOVA followed by Dunnett’s post hoc test).

To further investigate receptor involvement in CS-E-dependent responses, receptor interference experiments were performed in primary hippocampal neurons targeting CNTN-1 and integrin αVβ3. Consistent with previous reports [20], treatment with a CNTN-1 neutralizing antibody reduced CS-E-induced morphological changes (Figure 3A–C). Notably, inhibition of integrin αVβ3 using a functional blocking peptide [30] also attenuated these effects (Figure 3A–C). Although integrin αVβ3 has been primarily associated with CS-D-mediated neurite outgrowth [21], it has also been reported to interact with CS-E [31]. Together, these results suggest that integrin αVβ3 may cooperate with CNTN-1 in mediating CS-E-dependent signaling, potentially contributing to the more pronounced morphological effects observed under CS-E-enriched conditions compared with CS-D.

### 3.3. Involvement of CS-E-Dependent Src-Family Activation in a Polarization-Like Morphological Transition

**Figure 4:**
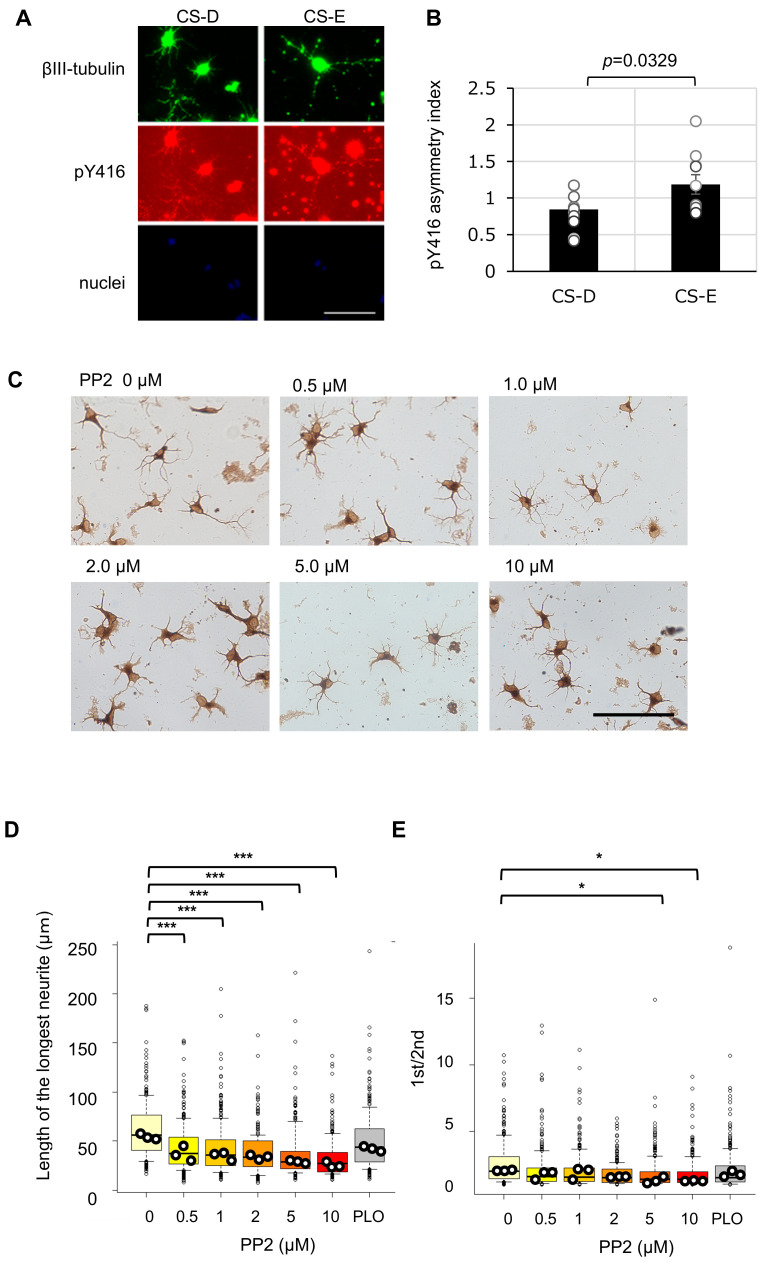
Association between chondroitin sulfate (CS)-E-mediated Src-family activation and polarization-like neuronal morphology. (**A**) Representative double immunostaining images of βIII-tubulin (green) and phosphorylated Src-family kinases (Tyr416; pY416, red) in hippocampal neurons cultured for 48 h on CS-D- or CS-E-enriched substrates. Nuclei were stained with DAPI (blue). Scale bar = 100 μm. (**B**) Quantification of pY416 asymmetry (pY416 asymmetry index), defined as the ratio of pY416 fluorescence intensity in the longest neurite to that in the second longest neurite in randomly selected neurons. Data are presented as mean ± SEM, with each dot representing an individual neuron (*n* = 10 per condition). Statistical significance was determined using Student’s *t*-test (*p* = 0.0329). (**C**) Representative images of hippocampal neurons cultured on CS-E-enriched substrates in the presence of the Src-family kinase inhibitor PP2 (0, 0.5, 1.0, 2.0, 5.0, or 10 μM). Scale bar = 100 μm. (**D**,**E**) Effects of Src-family kinase inhibition on CS-E-induced polarization-like neuronal morphology, quantified by the length of the longest neurite (**D**) and the ratio of the longest to second longest neurite ((**E**), 1st/2nd) under the conditions shown in (**C**). Data distributions are presented as box plots (boxes indicate the interquartile range [IQR], whiskers represent 1.5 × IQR, horizontal lines indicate the median, and small dots represent outliers). Larger dots represent mean value from each independent culture. A total of 100 neurons were analyzed per culture, with three independent cultures per condition. Statistical analyses were performed using mean values from independent cultures. * *p* < 0.05 vs. 0% PP2; *** *p* < 0.001 vs. 0% PP2 (one-way ANOVA followed by Dunnett’s post hoc test).

To investigate this mechanism, we performed immunocytochemical analysis to compare Src-family kinase activation in hippocampal neurons cultured on CS-D- or CS-E-enriched substrates. As shown in Figure 4A,B, neurons cultured on CS-E-enriched substrates exhibited significantly stronger pY416 immunofluorescence than those cultured on CS-D-enriched substrates. Notably, pY416 signals were preferentially localized to the longest neurite rather than the second longest, resulting in increased asymmetry of pY416 distribution (pY416 asymmetry index). These findings support the enhanced capacity of CS-E to promote Src-family kinase activation and suggest that CS-E-induced spatial compartmentalization of active Src-family kinases within the longest neurite contributes to the transition from a stage 2-like to a stage 3-like neuronal morphology.

Consistent with this interpretation, morphological indices of hippocampal neurons cultured on CS-E-enriched substrates—including the length of the longest neurite and the ratio of the longest to second-longest neurite—were reduced in a dose-dependent manner by the Src-family kinase inhibitor PP2 [32] (Figure 4C–E). These results further support a role for Src-family kinase activity in the acquisition of morphology associated with early neuronal polarization.

### 3.4. Effects of Fyn Activation on Neuronal Polarization-like Neuronal Morphology in N2a/CNTN-1 Cells

Given that highly sulfated CS subtypes are proposed to act as extracellular cues that activate Src-family signaling pathways, likely involving Fyn, we next examined whether modulation of Fyn activity alone influences polarization-like neuronal morphology. To this end, functional variants of Fyn kinase—wild-type (WT), constitutively active (CA), and dominant-negative (DN) forms [23,24]—were transiently expressed in N2a/CNTN-1 cells, and their morphological indices were compared. Western blot analysis confirmed expression of FLAG-tagged recombinant Fyn proteins with expected molecular weights for all constructs (Figure S3). To identify transfected cells, Fyn expression plasmids were co-transfected with a YFP vector, and analyses were restricted to YFP-positive cells.

**Figure 5:**
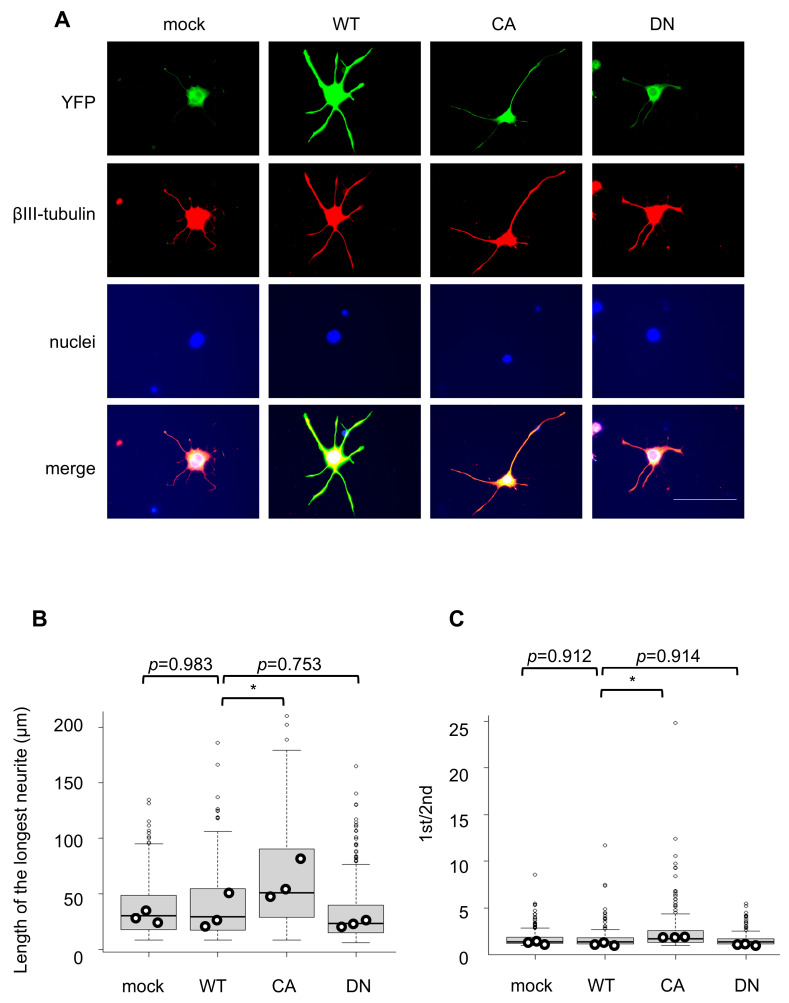
Forced activation of Fyn kinase facilitates polarization-like neuronal morphology in Neuro-2a/contactin-1 (N2a/CNTN-1) cells, even in the absence of chondroitin sulfate stimulation. (**A**) Representative immunofluorescence images of N2a/CNTN-1 cells co-electroporated with empty vector (mock) or expression plasmids for Fyn variants (wild-type [WT], constitutively active [CA], or dominant-negative [DN]) along with a YFP plasmid. After 72 h, the cells are immunostained for βIII-tubulin and YFP, and the nuclei are counterstained with DAPI. Scale bar = 100 µm. (**B**,**C**) Length of the longest neurite (**B**) and ratio of the longest to second longest neurite ((**C**), 1st/2nd) in YFP^+^ cells under each condition. Data distributions of individual cells are shown as box plots (boxes indicate the interquartile range [IQR], whiskers denote 1.5 × IQR, horizontal lines indicate the median, and small dots represent outliers). Larger dots represent the mean value from each independent culture. A total of 100 cells were analyzed per culture, and three independent cultures were included for each condition. Statistical analyses were performed using the mean values from independent cultures. * *p* < 0.05 vs. WT (one-way ANOVA followed by Dunnett’s post hoc test).

Expression of the CA Fyn mutant significantly increased morphological asymmetry indices, assessed using two complementary morphometric parameters, compared with WT Fyn (Figure 5A–C). In contrast, expression of the DN Fyn mutant reduced neurite extension from the cell body (Figure 5A) and significantly decreased the length of the longest neurite relative to WT (Figure 5B), without affecting the ratio of the longest to second longest neurite (Figure 5C). These results suggest that increased Fyn activity promotes morphological changes resembling those observed under CS-E-enriched conditions.

## 4. Discussion

In this study, focusing on the distinct morphological characteristics of hippocampal neurons cultured on CS-D- and CS-E-enriched substrates, we demonstrate that the transition from a stage 2-like to a stage 3-like neuronal morphology can be modulated by adjusting the mixing ratio of these two highly sulfated CS subtypes (Figure 1). We further show that this CS-mediated process is associated with intracellular regulation of Src-family kinase activity, likely involving Fyn. Although CS-D- and CS-E-dependent neurite outgrowth has been reported to involve distinct CS-binding receptors, both signaling pathways appear to converge on Src-family kinase activation [20,21]. In the present study, CS-E induced markedly stronger Src-family kinase activation than CS-D, suggesting that this difference contributes to the observed polarization-like morphological changes (Figure 2C,D).

Although CS-E has a higher negative charge density than CS-D based on its disaccharide composition (Table 1), the enhanced Src-family kinase activation is unlikely to be explained solely by nonspecific electrostatic effects. Rather, this difference may reflect the unique sulfation pattern of CS-E, characterized by 4,6-*O*-disulfated GalNAc residues, which can form specific recognition motifs for extracellular ligands and CS-binding receptors. Indeed, CS-E has been reported to interact with a broader range of extracellular molecules than CS-D [11,29,33], potentially facilitating the assembly of receptor-associated signaling complexes at the cell surface and thereby promoting more efficient activation of Src-family kinases.

Beyond these mechanistic insights, our findings may have broader implications. CS sulfation patterns may function as extracellular cues that regulate early stages of neuronal polarization. Alterations in CS sulfation patterns have been reported in conditions such as CNS injury and neurodevelopmental disorders [34,35,36]. However, whether such changes influence neuronal polarization in vivo or contribute to disease mechanisms remains unclear and warrants further investigation.

CNTN-1, identified as a CS-E-binding receptor, is a glycosylphosphatidylinositol (GPI)-anchored protein that lacks a cytoplasmic domain [37]. Nevertheless, it can organize functional membrane microdomains, such as lipid rafts, through *cis* interactions with transmembrane proteins, thereby facilitating intracellular signaling [37]. Through this scaffolding function, CNTN-1 may locally enhance Src-family kinase activation within specific neuronal membrane domains, including the prospective axon. Notably, the increased asymmetry of pY416 distribution observed on CS-E-enriched substrates (Figure 4A,B) likely reflects localized activation of Src-family kinases within neuronal processes where CNTN-1 may preferentially accumulate. These observations support a model in which CNTN-1 contributes to the spatial organization of signaling domains associated with axon-like features. Consistent with this, CNTN-1 has been reported to localize to the axon initial segment and contribute to the maintenance of axonal function [38]. Together, these findings suggest that CNTN-1 plays a role in the spatial regulation of Src-family signaling associated with axonal identity.

**Figure 6:**
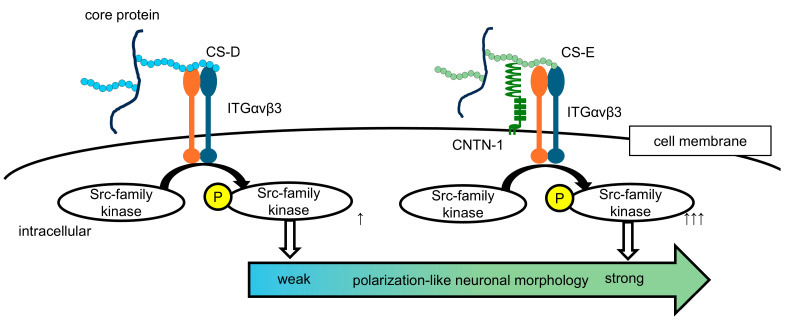
Proposed model of highly sulfated chondroitin sulfate (CS)-mediated regulation of polarization-like neuronal morphology. CS proteoglycans containing CS-D- and CS-E-enriched structures are proposed to function as environmental cues that influence polarization-like neuronal morphology. These CS chains may promote activation of Src-family kinases, with likely contribution from Fyn, through specific neuronal surface CS-binding receptors (integrin αvβ3 [ITGαvβ3] and/or CNTN-1), which are suggested to recognize distinct sulfation patterns. CS-E is proposed to elicit stronger activation of Src-family signaling than CS-D, thereby potentially facilitating stage 2-to-stage 3-like morphological transition, consistent with early axon-like features.

Our receptor interference experiments in primary hippocampal neurons indicate that both CNTN-1 and integrin αVβ3 contribute to CS-E-dependent morphological changes (Figure 3). Inhibition of CNTN-1 reduced CS-E-induced responses, consistent with previous reports identifying CNTN-1 as a CS-E-binding receptor [20]. In addition, blockade of integrin αVβ3 also attenuated these responses, consistent with its reported role in CS-D-dependent signaling [21]. These findings suggest that CS-E-dependent signaling is not mediated by a single receptor system but may involve multiple receptor components. One possible interpretation is that CNTN-1 and integrin αVβ3 act cooperatively to transduce CS-E-derived extracellular cues into intracellular Src-family kinase signaling (Figure 6). This framework may explain both the convergence of CS-D- and CS-E-dependent signaling on Src-family kinases and the stronger signaling and morphological effects observed under CS-E-enriched conditions. However, this receptor model remains hypothetical, as direct molecular interactions or cooperative mechanisms between these receptors have not yet been established.

Previous studies on neuronal polarity formation have not extensively addressed the involvement of Src-family kinases. However, studies in knockout mice indicate that Src-family kinases, including Src and Fyn, play critical roles in neuronal development and function [39,40,41,42]. In particular, in in vivo models of neuronal polarity formation—such as radial migration of developing cortical neurons—the transition from multipolar to bipolar morphology [4,5], corresponding to morphological polarization, is significantly impaired by Fyn knockdown [43]. Consistent with these findings, our study showed that overexpression of CA Fyn increased morphological asymmetry in N2a/CNTN-1 cells even in the absence of highly sulfated CS substrates (Figure 5A–C), suggesting that Src-family kinase activity may contributes to morphological changes associated with early stages of neuronal polarization, with Fyn likely playing a key role. Mechanistically, Src-family kinases may promote neurite elongation through downstream pathways regulating cytoskeletal dynamics, including actin filament organization and microtubule assembly during axon formation. Notably, impaired multipolar-to-bipolar transition has also been reported in the knockdown models of sulfotransferases involved in CS-D and CS-E biosynthesis—Ust and Chst15, respectively—with more pronounced effects observed upon *Chst15* knockdown [14,44]. These findings support our interpretation that CS-E-derived signaling may play a more prominent role than CS-D in promoting polarization-like morphological transitions (Figure 6).

Heparan sulfate (HS), another major sulfated GAG in the CNS, regulates neuronal development primarily by functioning as a co-receptor for extracellular ligands, including growth factors and axon guidance cues [45,46]. Through these interactions, HSPGs facilitate ligand–receptor interactions and modulate intracellular signaling pathways, including those involving Src-family kinases such as Fyn. However, in our previous study, neurite outgrowth of hippocampal neurons and N2a/CNTN-1 cells was not promoted on HS-coated substrates—similar to CS-A- or CS-C-coated substrates—under conditions designed to minimize the contribution of soluble factors [20]. These observations suggest that HS does not act as a permissive extracellular substrate for neurite extension but instead regulates neuronal polarity indirectly by modulating extracellular signaling environments through its co-receptor functions. In contrast, our results indicate that highly sulfated CS subtypes, particularly CS-D and CS-E, can directly influence polarization-like morphology when presented as extracellular substrates. Together, these findings suggest that distinct sulfated GAGs regulate neuronal polarity through complementary mechanisms as follows: HS primarily modulates signaling pathways as a co-receptor, whereas specific CS sulfation motifs act as instructive extracellular matrix cues.

In the developing CNS, the composition and sulfation patterns of CS chains are dynamically regulated in a spatiotemporal manner [13,14,15]. CS-E motifs have been detected in the developing mouse hippocampus and exhibit region-specific distributions [47,48]. Although their precise physiological roles remain unclear, these observations raise the possibility that locally enriched CS sulfation patterns function as extracellular cues influencing neuronal polarization in vivo.

Although N2a cells are widely used as a model system for studying neuronal differentiation and intracellular signaling, they are derived from neuroblastoma and do not fully recapitulate the properties of primary neurons [49,50]. In the present study, primary hippocampal neurons were used to assess neuronal morphological polarization, whereas N2a/CNTN-1 cells were employed as a complementary system to investigate CS-dependent Src-family signaling. Importantly, N2a/CNTN-1 cells represent an engineered model and should not be considered a direct surrogate for neuronal polarity mechanisms in primary neurons or in vivo contexts. Therefore, these findings should be interpreted with appropriate caution.

Despite these findings, several limitations warrant further investigation. First, neuronal polarization in this study was defined based on morphological criteria, and the molecular identity of the elongated neurite was not fully validated using definitive axonal markers. Accordingly, our data describe a polarization-like morphological transition rather than confirmed axon specification. Second, although our results support a model in which CS-binding receptors converge on Src-family kinase signaling, the precise molecular organization of these receptor complexes and their direct interactions remain to be elucidated. Future studies addressing these mechanistic aspects will be essential for a comprehensive understanding of how highly sulfated CS subtypes regulate neuronal polarity. Nevertheless, the consistent enhancement of Src-family kinase activation in response to CS-E supports its role as a potential upstream regulator of neuronal morphological polarization.

Finally, immunocytochemical analysis using an axonal marker indicated that, under CS-E-enriched conditions, the longest neurite tended to exhibit relatively stronger marker enrichment compared with other neurites, whereas such preferential enrichment was less apparent under CS-D-enriched conditions. Consistent with these observations, quantitative analysis of the SMI31 asymmetry index further supported a bias toward enhanced marker localization in the longest neurite under CS-E-enriched conditions. These observations are consistent with the interpretation that CS-E promotes early axon-like features. However, as this analysis does not definitively establish axonal identity, the findings should be interpreted as reflecting a polarization-like transition rather than fully established neuronal polarity.

## 5. Conclusions

This study demonstrates that highly sulfated CS subtypes, CS-D and CS-E, can influence polarization-like neuronal morphological transitions in vitro, potentially through activation of Src-family kinases via interactions with specific CS-binding receptors. These findings provide a foundation for further investigations into how extracellular matrix sulfation contributes to the regulation of neuronal morphology and, more broadly, neuronal circuit formation.

## Figures and Tables

**Table 1:** Disaccharide composition of chondroitin sulfate (CS)-D- and CS-E-enriched preparations used in this study.

Composition	Shark Cartilage CS-D	Shark Cartilage CS-E
mol % ^1^		
GlcA-GalNAc	0.70 ± 0.01	1.44 ± 0.32
GlcA-GalNAc(4-sulfate)	41.70 ± 0.18	22.09 ± 1.63
GlcA-GalNAc(6-sulfate)	37.24 ± 0.46	11.73 ± 1.19
GlcA(2-sulfate)-GalNAc(6-sulfate)	20.73 ± 0.69	ND ^2^
GlcA-GalNAc(4,6-disulfate)	0.26 ± 0.26	64.74 ± 2.65

^1^ Values are expressed as molar percentages of total disaccharides generated by digestion of each CS preparation with chondroitinase ABC and represent the mean ± standard error of the mean (SEM) for three independent determinations. ^2^ ND, not detected.

**Table 2:** Primers used for plasmid construction.

Construct		Primer Type	Sequence (5′–3′)
p3xFLAG/Fyn-WT		Forward	5′-GAATTC^1^GCCACCATGGGCTGTGTGCAATG-3′
		Reverse	5′-AAAGAATTCATAATGGGCTGTGTGCAATGT-3′
p3xFLAG/Fyn-CA	IM ^2^	Forward	5′-CGACAGAGCCCCAGTTCCAACCTGGT-3′
	IM	Reverse	5′-ACCAGGTTGGAACTGGGGCTCTGTCG-3′
	VR ^3^	Reverse	5′-ATCACTACTTGTCATCGTCATC-3′
p3xFLAG/Fyn-DN	IM	Forward	5′-AAGTAGCCATAATGACTCTTA-3′
	IM	Reverse	5′-TAAGAGTCATTATGGCTACTT-3′

^1^ Underlined sequences indicate restriction enzyme sites. ^2^ IM, internal mutagenic primers. ^3^ VR, vector region downstream of the multiple cloning site.

**Table 3:** Primers used for quantitative real-time reverse transcription-polymerase chain reaction.

Gene	Forward (5′–3′)	Reverse (5′–3′)
*Gapdh*	5′-CATCTGAGGGCCCACTG-3′	5′-GAGGCCATGTAGGCCATGA-3′
*Itgav*	5′-CCGTGGACTTCTTCGAGCC-3′	5′-CTGTTGAATCAAACTCAATGGGC-3′
*Itgb3*	5′-GAGCCCATTTTCTTCTCCCG-3′	5′-GCAACACCATGAATCCATCCC-3′

## Data Availability

The original contributions presented in this study are included in the article/supplementary material. Further inquiries can be directed to the corresponding author.

## Supplementary Materials

The following supporting information can be downloaded at https://www.mdpi.com/article/10.3390/cells15090747/s1, Figure S1: Uncropped Western blots for Figure 2C and Figure S3; Figure S2: Axon-like features of hippocampal neurons cultured on CS-D- and CS-E-enriched substrates.; Figure S3: Expression of FLAG-tagged Fyn kinase variants in Neuro-2a/contactin-1 (N2a/CNTN-1) cells.

## Abbreviations

The following abbreviations are used in this manuscript:

BDNF	brain-derived neurotrophic factor
CA	constitutively active
cDNA	complementary DNA
ChABC	chondroitinase ABC
Chst3	carbohydrate sulfotransferase 3
Chst11	carbohydrate sulfotransferase 11
Chst12	carbohydrate sulfotransferase 12
Chst15	carbohydrate sulfotransferase 15
CNS	central nervous system
CNTN-1	contactin-1
CS	chondroitin sulfate
DN	dominant-negative
GAG	Glycosaminoglycan
GalNAc	*N*-acetylgalactosamine
*Gapdh*	glyceraldehyde 3-phosphate dehydrogenase
GFP	green fluorescent protein
GlcA	glucuronic acid
GPI	Glycosylphosphatidylinositol
HS	heparan sulfate
HSP90	heat shock protein 90
IM	internal mutagenic
ITG	integrin
PG	Proteoglycan
N2a	neuro-2a
PBS	phosphate-buffered saline
PCR	polymerase chain reaction
PLO	poly-L-ornithine
PP2	4-Amino-5-(4-chlorophenyl)-7-(t-butyl)pyrazolo[3,4-d]pyrimidine
pY416	phosphorylated at tyrosine-416
qPCR	quantitative PCR
Ust	uronyl 2-*O*-sulfotransferase
WT	wild-type
YFP	yellow fluorescent protein
