# Supplementary material for: Sulfation of Chondroitin Sulfate Regulates Neuronal Morphology via Src-Family Signaling with Likely Contribution from Fyn
Source: Cells. 2026 Apr 22;15(9):747. doi: 10.3390/cells15090747 (PMC13162826; doi:10.3390/cells15090747)
Supplement: Supplementary file 1 [file cells-15-00747-s001.zip › cells-4195117-supplementary.pdf]

Supplementary Materials

Sulfation of Chondroitin Sulfate Regulates Neuronal Morphology via Src-Family Signaling with Likely Contribution from Fyn

Saya Kubosaka[1], Tadahisa Mikami[1], and Hiroshi Kitagawa[1,*]

[1] Laboratory of Biochemistry, Kobe Pharmaceutical University, Kobe, Japan; gd236021@st.kobepharma-u.ac.jp (S.K.); tmikami@kobepharma-u.ac.jp (T.M.)

[2] Correspondence: kitagawa@kobepharma-u.ac.jp; Tel: +81 78 441 7569

Figure S1: Uncropped western blots corresponding to Figure 2C and Figure S3.

Figure S2: Axon-like features of hippocampal neurons cultured on CS-D– and CS-E–enriched substrates.

Figure S3: Expression of FLAG-tagged Fyn kinase variants in Neuro-2a/contactin-1 (N2a/CNTN-1) cells.

Uncropped blots use in Figure 2C

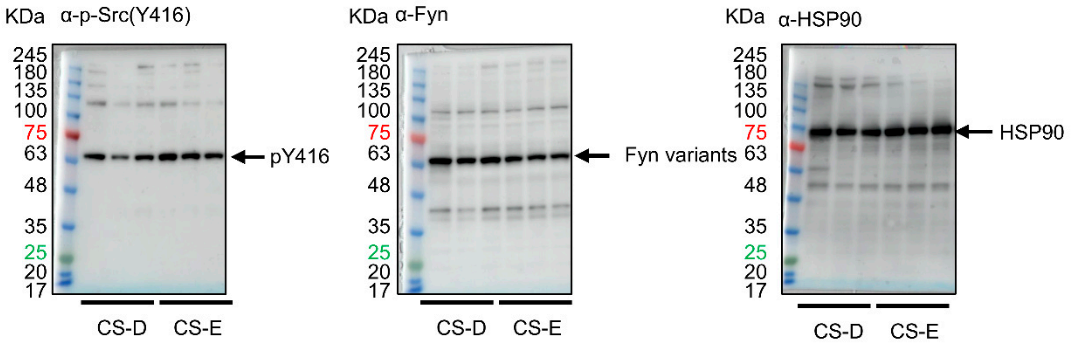

Uncropped blots Figure S2

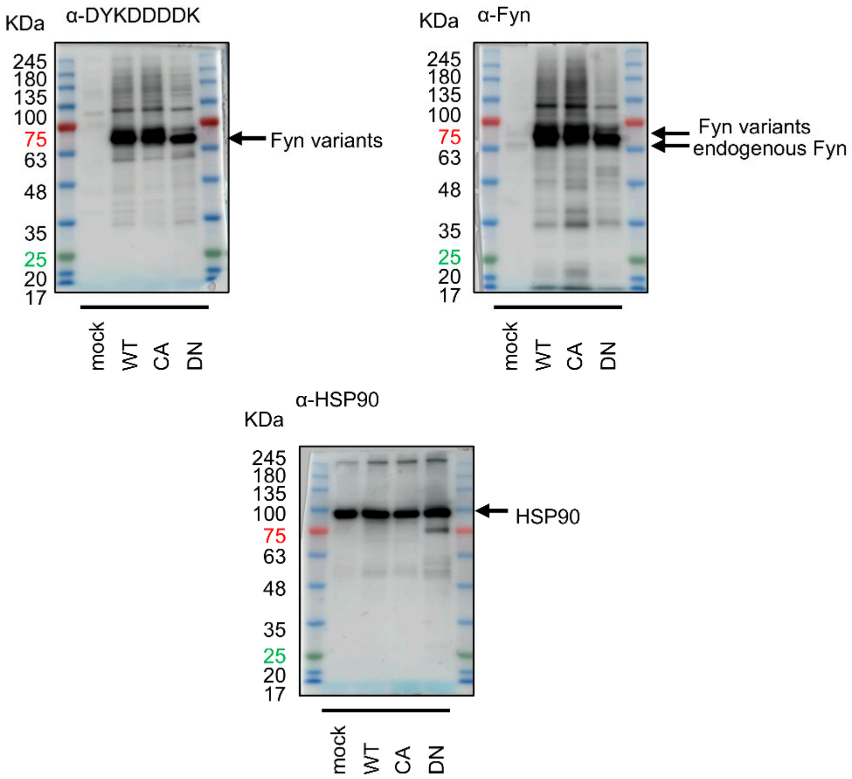

Figure S1: Uncropped western blots corresponding to Figure 2C and Figure S3.

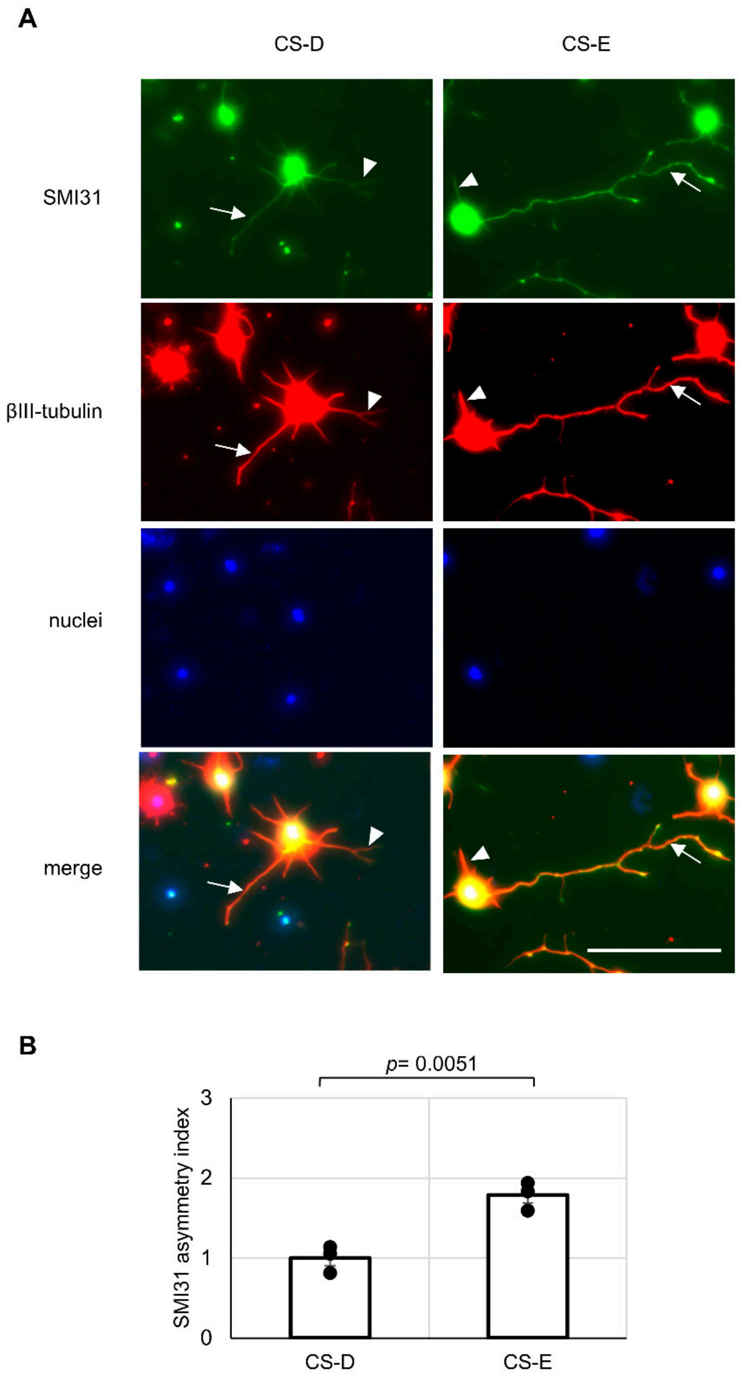

Figure S2: Axon-like features of hippocampal neurons cultured on CS-D– and CS-E–enriched substrates. Representative immunofluorescence images of hippocampal neurons cultured for 48 h on CS-D– or CS-E–enriched substrates. Cells were immunostained with antibodies against phosphorylated neurofilament H (SMI31; green) and βIII-tubulin (Tuj-1; red). Nuclei were stained with 4′,6-diamidino-2-phenylindole (DAPI; blue). Under CS-E–enriched conditions, the

longest neurite (arrow) frequently exhibits stronger SMI31 immunoreactivity, whereas under CS-D–enriched conditions, SMI31 signal appears more diffusely distributed without clear preferential localization. The second-longest neurite is indicated by arrowheads. Scale bar = 100 µm. (B) Quantification of the SMI31 asymmetry index, defined as the fluorescence intensity ratio between the longest and second-longest neurites. The ratio was calculated for individual neurons, and the mean value per culture was used for statistical analysis. A total of 15 neurons were analyzed per culture, with three independent cultures per condition. Data are presented as mean ± SEM, with each dot representing an independent culture (n = 3 per condition). Statistical significance was determined using Student's *t*-test ($p = 0.0051$).

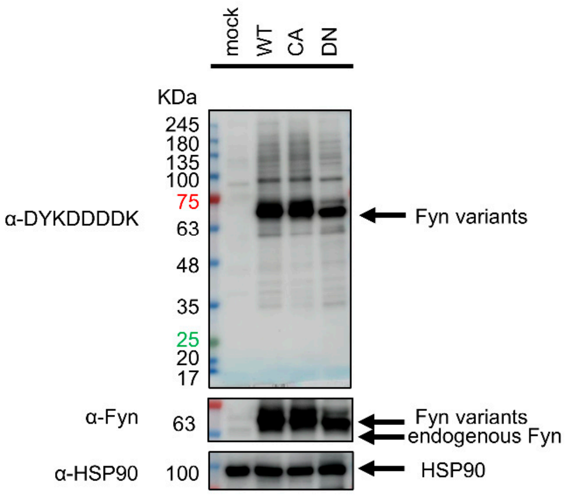

Figure S3: Expression of FLAG-tagged Fyn kinase variants in Neuro-2a/contactin-1 (N2a/CNTN-1) cells. Transiently expressed Fyn variants–wild-type (WT), constitutively active (CA), and dominant-negative (DN)–were detected by immunoblotting using anti-DYKDDDDK and anti-Fyn antibodies. Heat shock protein 90 (HSP90) was used as a loading control.
